# Estimation of muscular metabolic power in two different cross-country sit-skiing sledges using inverse-dynamics simulation

**DOI:** 10.1177/20556683221131557

**Published:** 2022-10-06

**Authors:** Marie Lund Ohlsson, Jonas Danvind, L Joakim Holmberg

**Affiliations:** 1 Swedish Winter Sports Research Centre, Department of Health Sciences, Mid Sweden University, Östersund, Sweden; 2 Sports Tech Research Centre, Department of Quality Management and Engineering Technology, Mid Sweden University, Östersund, Sweden; 3 Solid Mechanics, Department of Management and Engineering, Linköping University, Linköping, Sweden

**Keywords:** kinematics, kinetics, musculoskeletal modeling, para sports

## Abstract

The aim of this study was to estimate and compare the muscular metabolic power produced in the human body using musculoskeletal inverse-dynamics during cross-country sit-skiing. Two sitting positions were adapted for athletes with reduced trunk and hip muscle control, knee low with frontal trunk support (KL-fix), and knee high (KH). Five female national class able-bodied cross-country skiers performed submaximal and maximal exercise in both sitting positions, while recording 3-D kinematics, pole forces, electromyography and respiratory variables. Simulations were performed from these experimental results and muscular metabolic power was computed. The main part of the muscle metabolic power was produced in the upper limbs for both sitting positions, but KH produced more muscle metabolic power in lower limbs and trunk during maximal intensity. KH was also more efficient, utilizing less muscular metabolic power during submaximal intensities, relatively less power in the upper limbs and more power in the trunk, hip and lower limb muscles. This implies that sitting position KH is preferable for high power output when using able-bodied simulation models. This study showed the potential of using musculoskeletal simulations to improve the understanding of how different equipment design and muscles contribute to performance.

## Introduction

Cross-country sit-skiing (XCSS) is an endurance sport where athletes sit on a sledge mounted on a pair of skis and propel themselves forward by poles. The athletes in XCSS are classified (grouped) into locomotor winter (LW) classes: 10, 10.5, 11, 11.5, and 12. LW12 athletes have full control and functionality in the hip and trunk muscles and have full buttock sensibility. LW10 athletes have no control of the lower trunk or hip muscles and have no buttock sensibility.^1^ All cross-country sit-skiers compete in the same event and a factor-system is present to weigh the race time according to athletes’ classification.

The XCSS competition rules state that the sit-ski (sledge) cannot have any springs or flexible articulations, the buttocks shall be in contact with the seat during the whole race and the upper thighs must be strapped to the seat using non-flexible material.^2^ A general observation from world cup competitions is that athletes with full trunk and hip muscle control (e.g. LW12 athletes with leg amputation) sit knee-seated with their knees lower than their hips, while athletes with high impairment (LW10), such as reduced trunk and hip muscle control, sit with their knees higher than (or in level with) their hips.^3^ The intention is that the high knees should help the LW10 athlete, who has reduced trunk muscle function, to restrict the involuntary flexion-extension movement of the trunk and hips. With the reduced trunk and hip flexion the extension back to an erect starting position is easier. This issue is similar as in e.g. wheelchair racing, where trunk function is a central component and individual adjustment of seating position combined with strapping is important in order to maximize performance.^4^

Kinematics of XCSS athletes has been associated to the classes, showing that athletes with less impairment have larger trunk motion (defined as both hip and trunk motion).^3,5,6^ Also, the most impaired athletes, i.e. class LW10, have shown to initiate their poling phase earlier than others;^7^ this is likely because LW10 athletes need more time to generate propulsive power.

When abled-bodied participants have tested different sitting positions in a double poling ergometer, higher output speed was achieved when the knees were lower than the hips compared to when the knees were higher than the hips; the trunk was moving freely in all tested sitting positions.^8^ This study also showed that position of the legs impact trunk range of motion (ROM), i.e. a position with low knees showed larger trunk flexion-extension ROM than a position with high knees. Another study has also shown how restriction of trunk motion reduced power output for double poling in seated position.^9^ These studies both showed that a position with larger trunk flexion-extension ROM was associated with larger power output.

**Figure 1.**
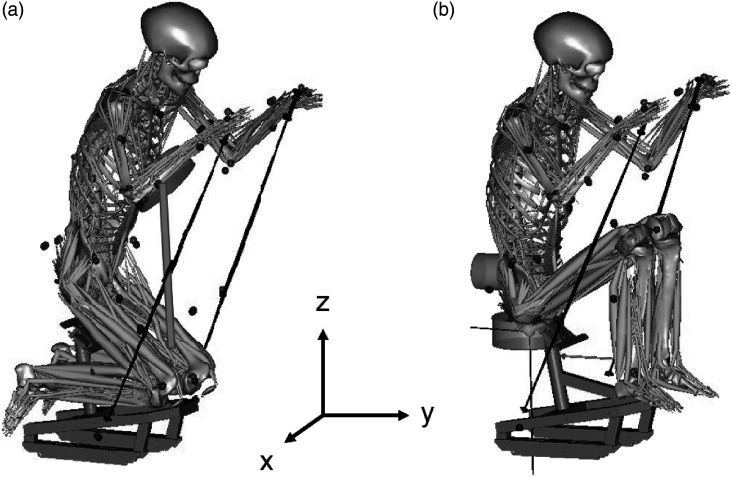
The simulation models with start of poling phase for: (a) the position with knees lower than hips (KL-fix), and (b) the position with knees higher than hips (KH). The cylindrical boxes are conditional contact points to the sit-ski, for KL-fix (frontal trunk support) and for KH (back support, the seat and the support in the fold of the knees).

Even though research indicates that larger trunk flexion-extension movement might be beneficial for performance, athletes with highly reduced trunk muscle control (such as LW10) cannot sit in a knee-seated position (as LW12 can). However, some athletes have successfully tried to adopt a knee-seated sitting posture with extra support around the hip.^6^ We have designed a new sit-ski sledge on which athletes with severely reduced trunk muscle control can sit in a knee-seated position. In this design, the trunk rests frontally against a support and is strapped with elastic bands to the support (Figure 1). This means that the trunk support restricts the flexion-extension movement of the trunk. In contrast, the common high knee sitting position for LW10 athletes restrict the flexion-extension movement of the trunk and hips because of the high knees.

In our earlier research,^10^ abled-bodied athletes tested both this new sitting position, knee-seated with frontal trunk support (KL-fix), and the common position for athletes with severely reduced trunk muscle control, where the knees are higher than the hips and the lower back rests against a support from the sledge (KH). These results showed how the KL-fix position was associated with reduced maximal power output, reduced spinal flexion and hip ROM, larger anaerobic metabolic rate, higher minute ventilation and impeded power output. These two sitting positions were also tested on the new sledge by an individual with complete spinal cord injury at thoracic vertebrae 4 (paralysis in lower trunk and legs). These tests also showed higher power output and larger flexion of hips and trunk during poling phase in KH compared to KL-fix.^11^ However, these studies could not make any conclusion regarding where the muscular work was produced in the body.

In different sports, different muscle groups have different importance for producing high power output. In able-bodied cross-country skiing double poling it has been shown that beyond shoulder and arm muscles also abdominal and hip-leg muscles are important for forward propulsion.^12,13^ It can be speculated that this is also the case for XCSS. One study of XCSS using electromyography (EMG) has shown preliminary results on activation in m. Erector spinae and m. Rectus abdominis activation for LW12 athletes while no trunk activation was recorded for LW10 athletes.^14^

According to the International Paralympic committee (IPC) classification code, classification provides a structure for competition and is performed to ensure that an athlete’s impairment is relevant to sport performance and that all athletes compete equitably.^1^ Evidence based classification is an important factor for creating fair competitions and training should not be a factor that can change classification of an athlete.^15,16^ In Para sports both the impairment and the equipment can affect how the muscular work is produced in the body. For classification there is an interest to increase understanding of where muscular work is produced in the body to understand how different impairments of muscular strength impact performance.^15^ Measurements of muscular activity is one option, however the drawback of using EMG is that each muscle needs to be measured separately which makes it hard to understand full-body muscle work. Also, the relationship between EMG and muscular force is less linear under dynamic conditions and there exist a time delay.^17^ Another option is musculoskeletal simulations, as for example inverse-dynamics simulations, which can estimate muscular work in a human model from measurements of kinematics and external forces. The drawback of this method is the approximations made to create subject-specific body models including the complex neuro-muscular system.^18^ However, today there is no other method of estimating distribution of muscle work in the whole body.^19^

The overall purpose of this study was to evaluate equipment design through musculoskeletal simulation using a computational method for muscular metabolic power. The specific aim of this study was to evaluate sit-ski design through estimating and comparing the muscular metabolic power produced in the whole human body using musculoskeletal inverse-dynamics able-bodied models of cross-country sit-skiing, for two sitting positions adapted for athletes with lower trunk and leg impairment. The hypothesis was that higher power output in maximal intensity exercise in KH compared to KL-fix is related to larger use of muscular metabolic power in trunk and lower limbs, while using similar muscular metabolic power in upper limbs.

## Methods

### Study overview

This study performed participant-specific musculoskeletal inverse-dynamics simulations using the Anybody Modeling System v 6.0 (Anybody Technology A/S, Aalborg, Denmark) of five of the ten athletes’ results from exercise tests in a previous study.^10^ The previous study recruited female able-bodied athletes competing in cross-country skiing or biathlon at national senior level. A subset of five athletes (62.6 ± 8.1 kg, 1.67 ± 0.05 m) used in this simulation study performed two familiarization sessions of 45 min exercise during the week before the experimental trials. The experimental trials comprised two sit-skiing ergometer tests (one test for each sitting position, KL-fix and KH (Figure 1)) in a randomized order and separated by at least 48 h. Each test was supervised by the same test leaders, carried out at the same time during the day, and included the same protocol and measurement methods; the only difference was the sitting position. Each sit-skiing ergometer test comprised a sub-maximal incremental component followed by a 3 min time trial (TT). The submaximal test commenced at 1560 W depending on participants’ fitness and included 4-7 stages of 3 min exercise and 1 min rest with increments of 7.5 W/stage. Participants were instructed to perform the highest mean power output during the TT.

Analysis was performed at three exercise intensities: submaximal stage 22 W (SUB2, low intensity below anaerobic threshold, blood lactate concentration [BLa^−^] 1.5 ± 0.4 mmol/l and respiratory exchange ratio (*RER*) 0.89 ± 0.05), the submaximal stage 37 W (SUB4, medium intensity around anaerobic threshold, [BLa^−^] 3.4 ± 1.7 mmol/l and *RER* 0.97 ± 0.05), and TT (maximal intensity).

This study had able-bodied participants to reduce the influence of different impairments on the results. For similar biomechanical characteristics, the study was limited to participants of one sex (female). The subset of five athletes were chosen as those who completed the fourth submaximal stage and for which high quality kinematics and kinetics data were recorded. The number of participants is justified due to the complexity of musculoskeletal simulations. All participants provided signed informed consent to participate in the study. The study was pre-approved by the Regional Ethical Review Board in Umea, Sweden (Dnr 2013–412–31M and 2015–74–32M).

### Measurements and equipment

A short overview of the methods of the measurements are given here, for a more extensive description see.^10^ This study performed measurements on the participants of height, weight, blood lactate concentration [BLa^−^], oxygen uptake, pole forces, kinematics and EMG. Thereafter simulations were performed of each participant using the measurements of height, weight, kinematics and pole forces. The sit-ski sledge, with the sitting positions KL-fix and KH (Ableway AB, Östersund, Sweden) was mounted on a ski-ergometer (ThoraxTrainer, ThoraxTrainer A/S, Kokkedal, Denmark). The participants were strapped to the sledge around ankles, knees, and pelvis. Additionally, in KL-fix, the thorax was strapped to the frontal support with elastic bands. Power output for each stroke was computed by the software of the ergometer using the known moment of inertia of the fly-wheel and the measured angular acceleration as a function of time (ThoraxTrainer ver 1.01, ThoraxTrainer A/S, Kokkedal, Denmark).

Respiratory variables, oxygen uptake and carbon dioxide production, were measured using a breath-by-breath method (Quark CPET, COSMED, Italy). In the sub-maximal levels, the mean of the third minute was utilized. For the TT the mean of 25 consecutive breaths with the largest total oxygen uptake was utilized. Blood samples were collected from the ear lobe and the blood lactate concentration [BLa^−^] was determined with Biosen C-line (EKF diagnostic GmbH, Magdeburg, Germany).

The aerobic metabolic power (*MP*_*ae*_) was computed from *oxygen uptake*, carbon dioxide production and gross energy expenditure using *RER* ≤ 1*.*00 as

(1) $${MP}_{ae}=\left(1.1\cdot RER+3.9\right)\cdot oxygen\;uptake\cdot 4184/60$$

The anaerobic metabolic power (*MP*_*an*_) was computed from [BLa^−^] by assuming that a 1 mmol/l increase in [BLa^−^] was equivalent to 3 mL/kg oxygen consumed^20^ and converted to metabolic power through equation (1). Total metabolic power (*MP*_*tot*_) is the sum of *MP*_*ae*_ and *MP*_*an*_.

**Figure 2.**
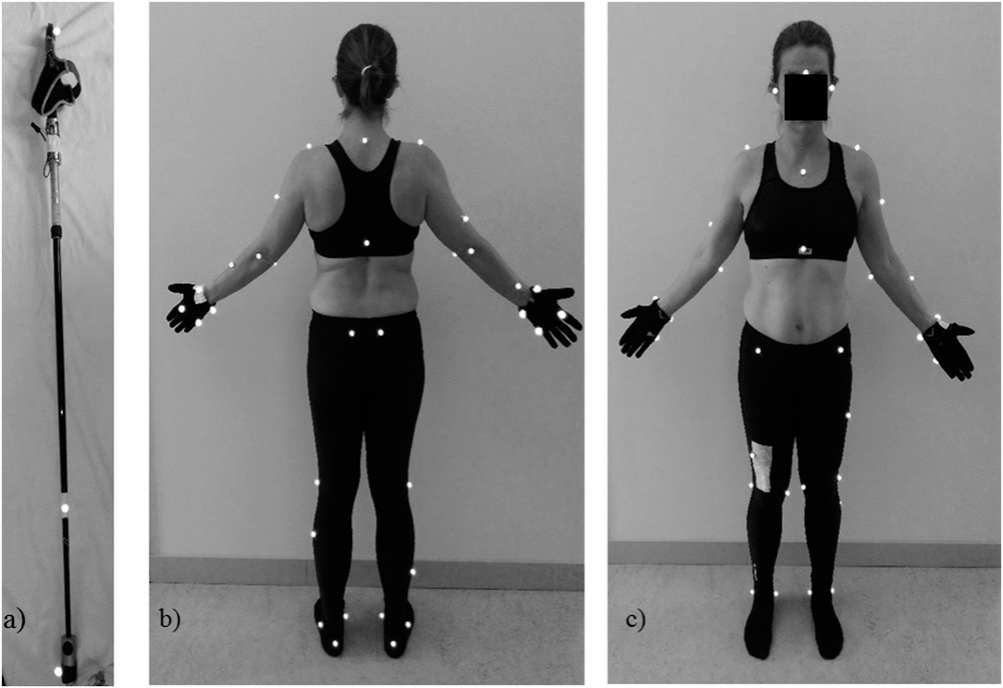
Marker placements for left pole (a) lower and upper limbs, pelvis, torso, head posterior view (b) anterior view (c).

Pole forces were measured axially at 250 Hz by linear strain gauge sensors, mounted between handle and pole, equipped with amplifiers (Biovision, Wehrheim, Germany). The horizontal pole force component (positive forward direction) was computed using the measurements of pole force and kinematics. Three-dimensional kinematics was recorded at 200 Hz with eleven Oqus3+ (Qualisys AB, Gothenburg, Sweden) cameras and the QualysisTrackManager software during the sit-ski ergometer test. A full-body marker set (modified plug-in-gait, www.vicon.com) comprising 49 markers (diameter 12 mm) were placed on the following locations (Figure 2): Tuber calcanei, lateral and medial malleolus, lateral and medial femoral epicondyle, lateral shank, lateral thigh, anterior and posterior superior iliac spine, (for KH anterior superior changed to iliac crest), xiphoid process, sternal notch, seventh cervical vertebra (C7), tenth thoracic vertebra (T10), acromion, lateral upper arm, lateral and medial epicondyle of the elbow, lateral forearm, radial styloid, ulnar styloid, metatarsal head 2 and 5, head (1 marker on glabella, 2 markers on temporal process of zygomatic bone), and poles (top, mid and tip).

Surface EMG was measured at 1000 Hz by TeleMyo 2400T G2 (Noraxon U.S.A. Inc., Scottsdale, USA) with electrodes with 20 mm diameter (Ambu blue sensor *N*, Ambu A/S, Ballerup, Denmark) and synchronized with kinematics and kinetics. Four muscles were measured on the right side of the body: m. Erector spinae longissimus (ESp), m. Rectus abdominis (RA), m. Latissimus dorsi (LD), and m. Triceps brachii caput laterale (TRI). For EMG normalization, maximum voluntary contractions were performed in 2 trials for each muscle separately. Electrode positions were marked on the skin by permanent marker and the electrodes were removed between the test sessions.

Kinematics, kinetics, EMG and simulations were analyzed over four poling cycles after 120 s in SUB2 and SUB4 and after 60 s in TT. The poling cycle started and ended when the pole tips were in their most forward position. The poling phase was defined as from the time when the pole tips were in their most forward position until their most backward position. The return phase was vice versa. Calibration of strain gauges in the Poles was made for 0, 5, 10, 15, and 20 kg and the signal was filtered using a 12 Hz, low-pass Butterworth filter. Joint angles were computed from marker data (filtered using a 10 Hz, low-pass Butterworth filter) through the parameter optimization procedure in the simulations. EMG data were processed in Matlab (R2015b, The Mathworks, Inc, Massachusetts, USA) and filtered by a Butterworth band-pass filter (50–300 Hz).

Participant-specific, inverse-dynamics musculoskeletal simulation models were built in the Anybody Modeling System v 6.0 (Anybody Technology A/S, Aalborg, Denmark) for both sitting positions, KL-fix and KH, over four poling cycles for SUB2 (after 120s), SUB4 (after 120s) and TT (after 60s). The AnyBody Modeling System is a software package that models the human body as a musculoskeletal rigid-body system with muscle actuators and formulates an inverse-dynamics static optimization problem to compute the muscle forces. The inverse problem was formulated as a static optimization problem where an objective function (a fifth order polynomial of the relative muscle forces) described how the redundant muscle recruitment problem was solved to the multibody system.^21^

The simulation models were modifications of the full-body MoCap model, available in the AnyBody Managed Model Repository v.1.6.3 (www.anybodytech.com) with added poles and sit-ski. The simulation models comprised 41 rigid segments and around 700 muscle actuators, for details see Figure 1 for a visual representation and^22^ for model details. The muscle actuators were of constant force model, i.e. maximum attainable force was constant over both length and speed and included no tendon unit, thus no activation dynamics, contraction dynamics or stretch-shortening effect. The body model and the sit-ski were mechanically connected to each other by both hard constraints (no motion) and soft constraints (motion).^23^ The hard constraints were defined for KH in ankles and for KL-fix in ankles, knees and seat. The soft constraints are visualized as cylindrical boxes in Figure 1, KL-fix: frontal trunk support, KH: knee support, seat, and backrest. Participants’ body height and weight were used to scale the respective simulation model’s height and weight. The segment masses and inertia properties were scaled according to.^24^ Kinematics data were low-pass filtered with 10 Hz and matched to the body models through an optimization procedure in the Anybody Modeling system.^25^ There, the models’ estimated segment masses and lengths were adjusted to match the actual dimensions of the participants. Scaling of the muscle strengths was made using the segment masses and lengths through the ScalingLengthMassFat function in the software. Overall, there were 30 unique simulations models, one for each sitting position KH and KL-fix, one for each participant and one for each of the three exercise intensities (SUB2, SUB4, TT). Validation and verification are important for the justification of musculoskeletal models.^26^ Validation studies of AnyBody Modeling simulation models have shown good agreement with EMG^27,28^ and with measured joint reaction forces.^29,30^

Muscular forces (*f*) were obtained through inverse-dynamics simulations in the AnyBody Modeling system. Muscular metabolic power was computed through

(2) $${mMP}_{tot}=\frac{\overset{n}{\underset{i=1}{\sum }}\int_{0}^{Cycle\;time}{mMP}_{i}dt}{Cycle\;time}$$

where *n* is the number of muscles and *mMP*_*i*_ was defined as

(3) $${mMP}_{i}=\left\{ \begin{array}{c} \;f_{i}\cdot v_{i}/1.25\;if\;v_{i}>0\\ -f_{i}\cdot v_{i}/0.25\;if\;v_{i}<0 \end{array}\right.$$

where *v*_*i*_ is the contraction speed and the difference in cost of eccentric and concentric work (row 1 and 2 in Equation (3)) was estimated based on.^31,32^ Positive contraction speed was defined as lengthening of muscle fiber.

The proportion of muscular metabolic power in a muscle group relative to total muscular metabolic power (*Rel mMP*_*group*_) was computed for three muscles groups: upper limbs (muscles with insertion on the arm), trunk (muscles in the trunk and neck without insertion on lower limbs or upper limbs), and lower limbs (muscles with insertion on the lower limbs).

### Statistical analyses

Statistical analysis was carried out using the Statistical Package for the Social Sciences (SPSS 22, IBM Corp., Armonk, New York, USA) and Office Excel 2013 (Microsoft Corporation, Redmond, Washington, USA). The level of significance was set at probability value (p) *<* 0.05. Physiological and biomechanical data were checked for normality with the Shapiro–Wilk analysis. When normality was violated, sitting positions were compared with Wilcoxon’s signed rank test for each exercise intensity. When normality was observed, data of the sitting positions were compared pair-wise for each exercise intensity with Student’s t-test. Two-way repeated-measures analysis of variances was used to analyze difference between the sitting positions and the exercise intensities SUB2, SUB4 and TT. If Mauchly’s test of sphericity was violated and the epsilon was *<* 0.75, the Greenhouse-Geisser correction was applied; while for epsilon *>* 0.75, the Huynh–Feldt correction was used.

## Results

In TT the power output and the *mMP*_*tot*_ were higher in KH than in KL-fix (*p <* 0.015 and *p* = 0.026). Considering the power distribution between muscle groups in TT, the relative muscular power for KH was higher in trunk and lower limbs than it was for KL-fix (higher *Rel mMP*_*trunk*_ for KH: main effect of position, F (1,4) = 20.3, *p* = 0.011, and higher *Rel mMP*_*lower limbs*_ for KH: main effect of position, F (1,4) = 10.91, *p* = 0.030). More results on the other two intensity levels are presented in Table 1.

**Table 1.** Results of kinematics, pole forces and metabolic power for the second and fourth submaximal stages (SUB2) and (SUB4) and maximal time trial (TT) for the sitting positions knee low (KL-fix) and knee high (KH). The asterisk (*) indicates a significant difference (*p* < 0.05) between KL-fix and KH.

	SUB2		SUB4		TT	
	KL-fix	KH	KL-fix	KH	KL-fix	KH
*Power output* (W)	22 ± 1	22 ± 1	36 ± 1	37 ± 1	48 ± 9	63 ± 13*
*mMP*_*tot*_ (W)	331 ± 21	318 ± 21	518 ± 62	465 ± 59*	791 ± 165	999 ± 84*
*mMP*_*upper limbs*_ (W)	314 ± 19	275 ± 24*	483 ± 43	405 ± 54*	728 ± 150	830 ± 80
*Rel mMP*_*upper limbs*_ (%)	95 ± 4	86 ± 4*	94 ± 4	87 ± 2*	92 ± 4	83 ± 2*
*mMP*_*trunk*_ (W)	11 ± 11	24 ± 8*	22 ± 19	35 ± 8	37 ± 17	92 ± 30*
*Rel mMP*_*trunk*_ (%)	3 ± 3	8 ± 3*	4 ± 3	8 ± 2	5 ± 2	9 ± 3*
*mMP*_*lower*_ _*limbs*_ (W)	7 ± 3	18 ± 7*	13 ± 9	26 ± 10	26 ± 19	77 ± 22*
*Rel mMP*_*lower limbs*_ (%)	2 ± 1	6 ± 2*	2 ± 1	5 ± 2*	3 ± 2	8 ± 3*
*MP*_*tot*_ (W)	420 ± 30	396 ± 11	749 ± 153	607 ± 80*	1429 ± 291	1450 ± 197
*Rel MP*_*ae*_ (%)	97 ± 3	99 ± 1	78 ± 9	89 ± 8*	60 ± 5	58 ± 5
*Rel MP*_*an*_ (%)	3 ± 3	1 ± 1	22 ± 9	11 ± 8*	40 ± 5	42 ± 5
*Cycle time* (s)	1.59 ± 0.03	1.64 ± 0.02	1.43 ± 0.03	1.55 ± 0.02	0.93 ± 0.02	0.93 ± 0.03
*Cycle length* (m)	0.99 ± 0.03	0.97 ± 0.03	1.08 ± 0.04	1.05 ± 0.03	0.93 ± 0.02	0.98 ± 0.03
*PPrel* (%)	49.9 ± 1.1	49.8 ± 1.3	50.1 ± 0.9	46.8 ± 1.1	59.4 ±1	56.1 ± 1.4*
*Peak pole force* (*N*)	84.9 ± 7.9	73.0 ± 3.7	118.4 ± 8.7	104.3 ± 7.8	121.5 ± 7.2	130.6 ± 3.9
*Mean pole force* (*N*)	29.8 ± 2.0	25.9 ± 1.7	38.3 ± 2.5	31.1 ± 1.4*	44.8 ± 2.1	44.9 ± 3.9
*Peak pole horizontal force* (*N*)	62.2 ± 5.8	58.4 ± 7.3	83.8 ± 11.7	81.3 ± 12.1	82.1 ± 8.2	96.8 ± 5.8*
*Mean pole horizontal force* (*N*)	22.0 ± 1.5	20.2 ± 2.3	27.0 ± 2.6	23.6 ± 3.3*	31.6 ± 3.8	33.6 ± 2.5
*Hip flexion ROM* (°)	4 ± 2	10 ± 5*	7 ± 3	12 ± 6*	9 ± 4	14 ± 6
*Hip flexion max* (°)	59 ± 7	109 ± 3*	59 ± 8	110 ± 5*	60 ± 6	114 ± 4*
*Spine flexion ROM* (°)	10 ± 7	20 ± 5*	15 ± 6	24 ± 5*	15 ± 4	22 ± 7*
*Spine flexion max* (°)	29 ± 10	55 ± 5*	32 ± 11	60 ± 5*	37 ± 11	69 ± 18*

**Figure 3.**
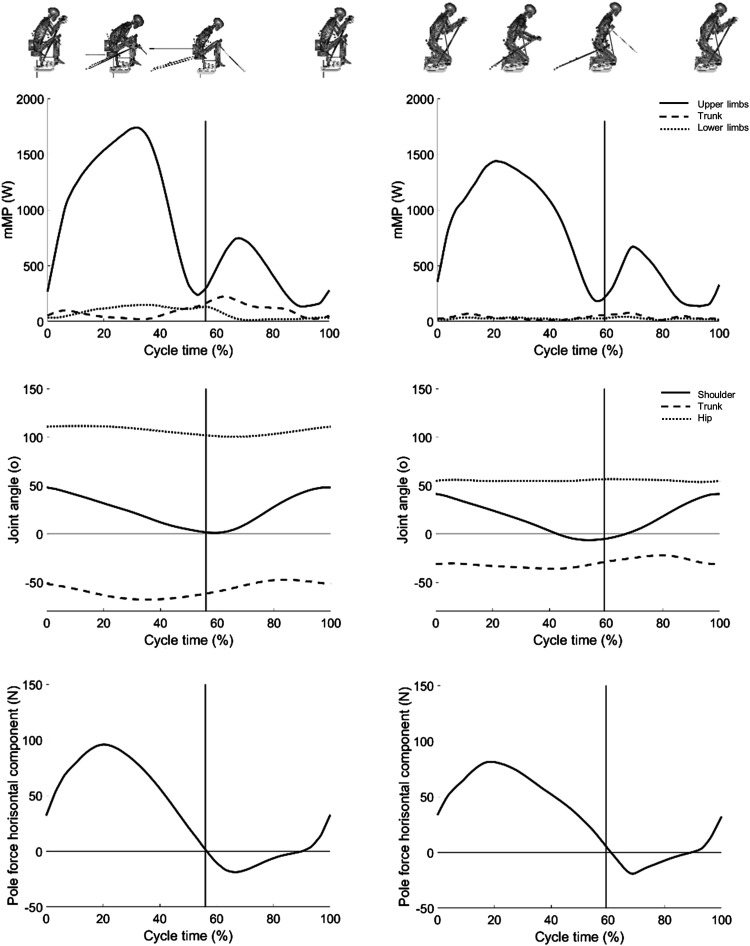
Kinematics and kinetics mean curves (*n*=5) over the cycle (0–100%) for the maximal exercise intensity test TT, for knee high sitting position (KH), left column, and knee low sitting position (KL-fix), right column. Upper row muscular metabolic power (*mMP*) for upper limbs (solid lined), trunk (dashed line) and lower limbs (dotted line). Mid row joint angles for shoulder flexion-extension (solid line), trunk flexion-extension (dashed line) and hip flexion-extension (dotted line). Lower row pole force horizontal component. Vertical lines show pole tips in their furthest back position. Definitions of joint angles, in anatomical position, are: shoulder = 0^◦^ (flexion positive, extension negative), trunk flexion = 0^◦^ (angle between pelvis and trunk in the sagittal plane with flexion negative), and hip = 0^◦^ (flexion positive).

The graph of the instantaneous *mMP* for the muscle groups lower limbs, trunk and upper limbs are shown in Figure 3. The main part of the muscle metabolic power was produced in the upper limbs for both sitting positions. During TT more muscle metabolic power was produced in both upper limbs, lower limbs and trunk for KH. The lower limb muscles performed the most during poling phase while the trunk muscles performed the most during the end of the poling phase and during the return phase.

**Figure 4.**
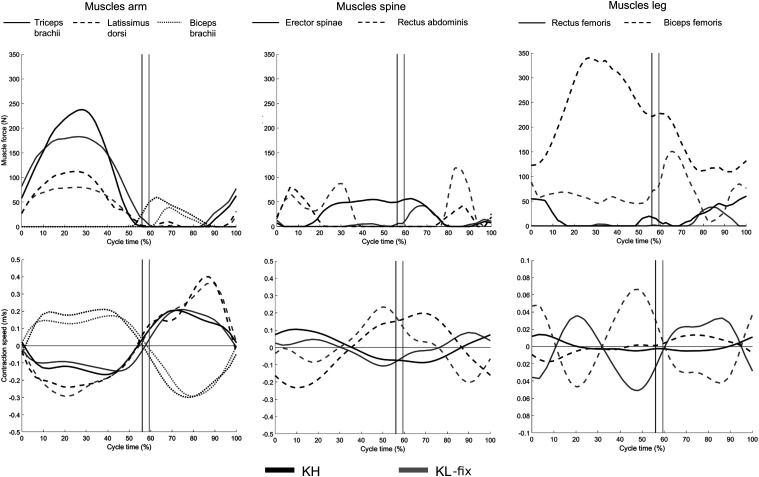
Muscle force (*N*), upper row, and contraction speed (m/s), lower row, for selected muscles for one participant in maximal exercise intensity, TT, over the cycle time (0–100%) for knee-high sitting position (KH, black) and knee low sitting position (KL-fix, grey). Presented muscle parts in upper limb are triceps long head part 1, biceps brachii caput breve, latissimus dorsi part 5, in trunk is rectus abdominis, erector spinae part T11-sacrum, and in lower limb are rectus femoris, biceps femoris long head part 1. Positive contraction speed is lengthening of muscle. Vertical lines show pole tips in their furthest back position.

For the upper limbs there were double peaks present, one during poling phase and one during return phase. Force and contraction speed for the muscles producing the most muscular metabolic power for one participant are shown in Figure 4. This show that triceps brachii and latissimus dorsi produced the muscular metabolic power during poling phase and biceps brachii during return phase. Muscle metabolic power was produced during eccentric muscle contraction in the end of poling phase for biceps brachii and in the end of the return phase for latissimus dorsi and triceps brachii.

For the trunk most muscle work was produced in KH (Figure 4). Here the main muscles that produced muscular metabolic power were rectus abdominis and erector spinae. Rectus abdominis performed muscular metabolic power in the beginning of the poling phase and erector spinae produced muscular metabolic power from the mid of the poling phase to mid of return phase. Both these muscles showed eccentric action at the start for a short time before displaying concentric action.

For the lower limbs there were low contraction speed and most muscular metabolic power was produced in sitting position KH. The main muscles producing muscular metabolic power in both sitting positions were hamstrings (all of the parts but mostly biceps femoris long head), hip adductors and the hip flexor rectus femoris.

Cycle time and cycle length showed no difference between sitting positions. The relative time of the poling phase was shorter for KH in TT (*p <* 0.050) and in SUB 4 for all participants, but not significant (*p* = 0.111), (Table 1).

Total metabolic power was higher for KL-fix compared to KH in SUB4, (*p* = 0.04) and showed no difference in SUB2 and TT (*p >* 0.050) (Table 1). *Rel MP*_*an*_ was higher (*Rel MP*_*ae*_ was lower) in KL-fix compared to KH in SUB4 (*p* = 0.002) and showed no difference between sitting positions in SUB2 and TT (Table 1).

**Figure 5.**
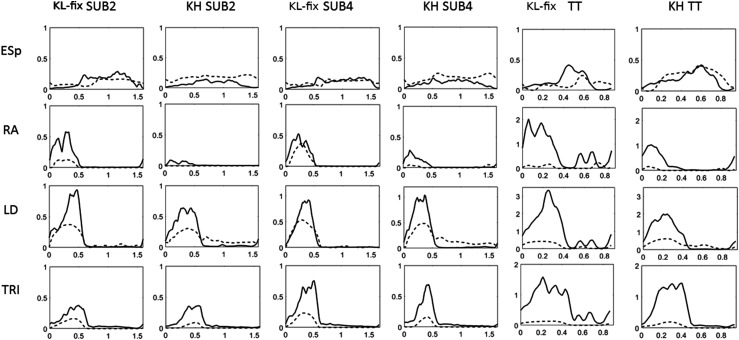
Normalized EMG (solid line) and simulation results of normalized force (dashed line) of one participant for four muscles (m. Erector spinae longissimus (ESp), m. Rectus abdominis (RA), m. Latissimus dorsi (LD), m. Triceps brachii caput laterale (TRI)) in sitting position knee low (KL-fix) and knee high (KH) for submaximal intensities SUB2, SUB4 and maximal time-trial (TT).

Peak pole forces (Table 1) showed no difference between sitting positions while the horizontal component of peak pole force was larger in KH during TT (*p* = 0.012). Mean pole force and the horizontal component of the pole force (Table 1) were lower for KH during SUB4 (*p* = 0.010 and *p* = 0.017), while no difference was observed during SUB2 and TT. The KL-fix position resulted in a less flexed hip and less flexion of the trunk, while KH revealed a slightly larger ROM in hip together with larger trunk flexion and trunk ROM (Table 1). A visual comparison of EMG and simulated muscle forces (normalized) for one representative participant is presented in Figure 5.

There was an interaction effect between metabolic power and muscular metabolic power for both sitting positions (KL-fix: F (2,8) = 20.12, *p <* 0.001, KH: F (2,8) = 19.35, *p <* 0.001); *MP*’s were higher than corresponding *mMP*’s and the differences were increasing with increasing power output (higher exercise intensity).

## Discussion and implications

This study showed that musculoskeletal simulations provide information of the muscular metabolic power produced in the body. Using able-bodied simulation models the main finding was that the hypothesis was confirmed; higher power output in TT for sitting position KH was related to larger muscular metabolic power in trunk and lower limbs. The results also showed that KH, compared to KL-fix, had lower total muscular metabolic power during submaximal intensity (indicating better efficiency) and higher muscular metabolic power during maximal intensity (indicating a higher propulsive power). The main part of the muscle metabolic power was produced in the upper limbs for both sitting positions. In comparison, KH produced larger amount of relative muscular metabolic power in the muscles around the trunk, hips and lower limbs, while KL-fix produced larger amount of relative muscular metabolic power in the upper limbs. The comparison between simulation results of muscular force and EMG showed similarities, while the comparison between muscular metabolic power and metabolic power revealed a difference that increased with increasing exercise intensity.

In sports as XCSS both aerobic and anaerobic energy sources are important. The comparison between the muscular metabolic power (computed from the simulations) and the metabolic power (computed from the physiological measurements) showed lower values of muscular metabolic power than metabolic power. Because these two quantities are results from different measurements and approximations, this is not surprising. Interestingly, the difference between the metabolic power and the muscular metabolic power increased when power output increased. One possible explanation for this increasing difference is that the metabolic power (based on measurements of oxygen uptake and blood lactate concentration) capture both fatigue and anaerobic metabolism. Muscle force created by anaerobic metabolism costs more energy than force created by aerobic metabolism;^33^ therefore the metabolic cost increases more with increased intensity than the muscular metabolic cost. The simulations included a linear relation between muscular metabolic power and muscular force (Equation (3)), meaning that there is no difference in metabolic cost for muscle force at low or high intensity exercise, which is not true in reality but the onset of anaerobic metabolism is both individual and trainable. It has also been shown that gross efficiency (power output divided by aerobic metabolic power), and thereby the estimation of aerobic metabolism, is related to fatigue.^34^ This demonstrates that the simulations capture the efficiency of the technique without the involvement of different energy sources and fatigue, which are parameters affected by training.

Research of technique analysis in general have shown that muscle activation and segment motion initiation in proximal to distal order is related to high power output.^35^ In seated double poling the proximal to distal sequencing chain has been identified as first trunk and hip motion, followed by shoulder and elbow motion.^11,14,36^ The current study showed that the KH position compared to KL-fix, had larger motion of hips and trunk, i.e. more motion was generated in the proximal part of the segment chain (Figure 3). These results agree with other studies of able-bodied athletes performing seated double poling,^37^ which showed the importance of trunk and hip muscle activation on high power output. Also, studies on Para athletes in XCSS have also shown an association between larger ROM in hip and trunk with higher performance in a race.^3,5^ However, in those studies, where only kinematics were analyzed and not kinetics, it is not clear that the increased motion in hip and trunk contributed to increased forward propulsive power output. Instead, the musculoskeletal simulations in the current study reveal this relation between the muscular metabolic power and forward propulsive power output; the muscular metabolic power was larger in the lower limbs and trunk and smaller in the upper limbs for the KH position compared to the KL-fix position.

The details of the muscle contraction coordination pattern of the current study showed differences between KH and KL-fix. For the lower limbs in KH, the hip extensors and adductors produced the main metabolic power. The hamstrings performed concentric action when flexing the trunk (from 95% to 40% of cycle time) and causing a small backward rotation of the pelvis. The hamstrings then performed eccentric action to reduce speed of forward rotation of pelvis and assisting erector spinae to lift the trunk back up to erect posture again. Rectus abdominis performed eccentric action to reduce joint angle speed of the trunk extension (from 75% to 85% of cycle time). The arm extensors also acted eccentric to reduce the shoulder flexion speed in the end of the return phase, and at the start of poling phase the arm extensors and rectus abdominis worked concentric. The arm extensors produced muscular metabolic power during the whole poling phase while the trunk flexors stopped working after 20% of poling time. This indicates how the abdominal muscles are present in the beginning of poling phase but thereafter the trunk extensors need to reduce the forward flexion of trunk and raise the trunk before the next poling phase. This indicated a proximal to distal order of activation: (1) the first preparation (20–85% of cycle time) starting with hip and trunk extensors breaking the forward flexion of hip and trunk and lifting the trunk up to erect posture; (2) the second preparation (55–90%) with shoulder flexors lifting the arms while trunk flexors and shoulder extensors acting eccentric before poling action; (3) start of poling action (0–25%) with muscular metabolic power produced in shoulder extensors (concentric action of latissimus and triceps), trunk flexors (concentric action of rectus abdominis) and hip muscles (concentric action of biceps femoris and some eccentric action of rectus femoris); and (4) end of poling action (25% to end of poling phase) when shoulder extensors continue to produce work during muscle shortening.

The detailed description of muscle action showed a complex muscle activation pattern. Because poling is a cyclic movement, the action of getting back to starting position (the erect posture at start of poling phase) is as important as performing the poling phase. The simulation showed that the return to starting position started with the eccentric action of hip extensors and trunk extensors, adding concentric action of shoulder flexors, to perform a quite stable hip and a trunk extension and shoulder flexion. In the end of that sequence rectus abdominis and shoulder extensors acted eccentric. The poling action then started with a combined concentric action of hip extensors and rectus abdominis flexing the trunk and concentric action of shoulder extensors extending the arms. So, therefore we interpret this movement of KH to involve two parts of a two-step proximal-distal sequence during the poling cycle. Both parts, the getting back to starting position and the poling action, involves first a combined hip and trunk action and thereafter the shoulder action.

Comparing the KH motion to the KL-fix motion showed how the action of both trunk and lower limb muscles are reduced. Therefore, the KL-fix motion was interpreted as a shorter sequence of proximal to distal activation because of reduced lower limb and trunk muscle metabolic power. The reduced power production in trunk and lower limbs and the reduced motion of the trunk was interpreted as the reason for lower horizontal pole force, power output and lower muscle metabolic power production in both the shoulder extensors (during poling phase) and flexors (during return phase). This means that relatively more muscular metabolic power was produced in the proximal part of the segment chain (lower limbs, hips and trunk muscles) for the sitting position KH, implying a more powerful technique that produced higher forward propulsive power output. This is in line with the discussion of other experimentally based studies showing higher forward propulsive power output with trunk and upper limb powered motion compared to upper limb powered motion only, in both double-poling^9^ and wheelchair propulsion.^38^

As mentioned in Methods, AnyBody Modeling simulation models have shown good agreement with EMG in earlier studies. The visual comparison presented in the current study between EMG and relative muscular force, using a single participant (Figure 5), showed similarities in onset and offset for RA, LD and TRI. Onset of the EMGs were slightly before relative muscle forces because the simulation models did not account for activation dynamics.^26^ Amplitude comparisons were not possible because strength of the body models was scaled from body size and not matched to the real strength of the participants. Moreover, EMG does not reflect the real strength because muscle activity is not linearly related to muscle force in non-isometric contractions.^39^ It is also important to point out that a muscle that contracts use energy even though there is no length change of the muscle, which means no work is produced. In this case EMG can show activity when no external work is performed, due to the reason that isometric contraction is not defined as mechanical work. All muscles measured with EMG had a non-zero contraction speed during the simulation.

The current study has shown how two different sitting positions change the muscle metabolic power production in the body during seated double poling using simulation models of able-bodied athletes. Understanding how different equipment and muscles contribute to performance is important for Para sports classification and Para sports competition rules.^15^ But it is a difficult task to design a study answering the question of how impairment affects performance. Often this is made with Para athletes performing a maximal intensity short-term trial while measuring biomechanical parameters.^15,40^ With such a study design it is hard to control for participant fitness level (of both strength and endurance capacity) and to distinguish between the impact of impairment and equipment (e.g. sitting position). Instead, musculoskeletal simulations are performed on a model of the human body and that leads to several limitations such as the level of detail of the body model, e.g. muscle model, number of muscles, approximations of the joint motions and the muscle recruitment algorithm etc. On the other hand, the advantage of computer simulations is that the simulation model has a constant fitness level, does not fatigue, parameters of all muscles are computed, and impact of equipment and impairment is possible to distinguish between. Therefore, musculoskeletal simulations could be a complement for Para sports classification research in order to understand how different equipment (e.g. sitting position) and impairments (active muscle groups) impact propulsive power output and thereby also performance. In a similar way, simulations may also contribute to equipment design to improve performance and simultaneously trying to provide fair and equal conditions. How these factors are balanced may vary from time to time and should be agreed upon by the parties in Para sports. Simulation is a tool to provide additional information in advance for understanding, developing and decision-making in equipment design.

The results imply that it is important for XCSS athletes to choose a sitting position and a technique that enhance muscle metabolic power in as large muscle mass as possible, such as using the lower limbs and trunk muscles to enhance power output. Of course, this depends on the type of impairment if it is possible to engage these muscles. In addition, achievement of a proximal to distal muscle activation sequence enhance power output; first the thighs, hips and trunk muscles and thereafter the shoulder and arm muscles. The KH position is the preferable position for able-bodied athletes, compared to the KL-fix position, because KH enables higher maximal propulsive power output and shows a tendency for higher efficiency.

## Conclusions

This study compared two sitting designs that are possible to use for athletes with impairments in the trunk and lower limbs, using simulation models of able-bodied athletes. The study concludes that KH is preferable compared to KL-fix for forward propulsive power output; KH produced larger total muscle metabolic power in the body during maximal intensity through larger muscle metabolic power production in the lower limbs and trunk and thereby contributed to power output more than KL-fix. The sitting position KH was also more efficient, utilizing less muscular metabolic power during submaximal intensities, and the relative muscular metabolic power was larger in the lower limbs and trunk muscles but lower in upper limb muscles. The hypothesis was thus mostly confirmed and deviating only regarding upper limb power.

This is a contribution to technique analysis, answering why forward propulsive power output and thereby also performance was better in position KH by showing how different muscle groups contributed to power output, i.e. a predictive technique analysis of the human-equipment interaction. This study also showed the potential of using musculoskeletal simulations to improve the understanding of how different sitting positions, equipment and muscles contribute to performance, which is an important question for Para sports classification research and optimal sit-ski design.
